# Dietary fish oil improves intestinal barrier function and intestinal microbiota composition and reduces systemic inflammation in a mouse model of moderate acute malnutrition

**DOI:** 10.1017/jns.2026.10114

**Published:** 2026-06-15

**Authors:** Grace E. Patterson, Elvia Yaneth Osorio, Sara M. Dann, Peter C. Melby

**Affiliations:** 1 Microbiology and Immunology, The University of Texas Medical Branch at Galveston, Galveston, USA; 2 Internal Medicine, The University of Texas Medical Branch at Galveston, USA

**Keywords:** Childhood malnutrition, Fish oil, Inflammation, Intestinal barrier, Microbiota

## Abstract

Acute malnutrition (wasting) remains a global public health problem. Ready-to-use therapeutic foods (RUTF) and supplemental foods (RUSF) for treatment of severe acute malnutrition (SAM) and moderate acute malnutrition (MAM), respectively, come in the form of macro- and micronutrient-dense pastes. These lipid-dense treatments provide the energy and nutrients needed to support growth but have significant rates of relapse. Their role in reversing pathophysiological contributors to malnutrition, such as intestinal barrier dysfunction, intestinal dysbiosis, and systemic inflammation have not been evaluated. Traditional lipid-dense RUTFs and RUSFs are rich in pro-inflammatory omega-6 polyunsaturated fatty acids (PUFAs) but low in anti-inflammatory omega-3 PUFAs, which could fuel malnutrition-related pathological inflammation. We reasoned that reduced dietary omega-6 (*n*-6) PUFAs and increased dietary long-chain omega-3 (*n*-3) PUFAs found in fish oil would reduce malnutrition-related pathological inflammation. In an established mouse model of MAM, we observed that altering the dietary *n*-3/*n*-6 PUFA ratio through replacing dietary corn oil with fish oil improved the diversity and composition of the caecal microbiota, improved intestinal mucosal barrier and immune defence, reduced translocation of bacteria and bacterial lipopolysaccharides (LPS), and dampened systemic inflammation. Furthermore, dietary fish oil protected against weight loss upon systemic challenge with bacterial LPS. The anti-inflammatory effects of dietary fish oil did not compromise host defence against challenge with the intestinal pathogen, *Citrobacter rodentium*. Collectively, these results suggest that dietary fish oil blunts inflammation that contributes to the pathogenesis of MAM. Inclusion of fish oil in dietary interventions may be beneficial in prevention or reduction of malnutrition-associated inflammation.

## Introduction

The global burden of malnutrition is staggering. In 2024, 42.8 million children had acute malnutrition (wasting), of which 71% had moderate acute malnutrition (MAM).^(1)^ Across multiple countries and cultures, the degree of undernutrition directly correlates with childhood mortality.^(2)^ A third of childhood deaths worldwide are attributed to the synergy between malnutrition and infection.^(3)^ Sustained prevalence of malnutrition will continue to drive childhood mortality, disability-adjusted life years (DALYs), and decline of national GDP, especially in Asia and Africa.^(3,4)^

Over the past decade, community-based therapy with a ready-to-use therapeutic food (RUTF)^(5,6,7)^ and ready-to-use supplemental food (RUSF)^(8,9,10,11,12,13)^ has changed the treatment of childhood severe acute malnutrition (SAM) and MAM, respectively. These formulations have high lipid content to make them energy-dense and high protein and are fortified with vitamins and trace elements.^(14)^ They are easy to use in resource-limited settings, as they do not require reconstitution (with potentially contaminated water) or refrigeration. Short-term interventions with RUTF and RUSF are effective in promoting growth,^(11)^ but treatment failure and relapse remain a significant problem.^(15,16,17,18)^ Analysis of pooled data from >20,000 severely malnourished children showed that RUTF had a failure rate of >20%. ^(15)^ Furthermore, despite success in growth recovery, the risk of infectious diseases remained high^(19)^ as did the overall mortality.^(16,19)^ These data suggest that current treatments do not adequately address the pathophysiological drivers of acute malnutrition.^(20,21,22,23,24)^

We determined previously that children with MAM, but without evidence of concurrent infection, had low-grade systemic inflammation, impaired intestinal mucosal barrier function, increased serum LPS (endotoxaemia), and an exaggerated response to inflammatory stimuli.^(22)^ We recapitulated these findings in a mouse model that mirrored the growth metrics of children with MAM.^(25,26,27,28,29)^ In this model, in which corn oil was the source of lipid in the control and nutrient-deficient diets, we found that altered intestinal microbiota composition and impaired intestinal barrier function drive endotoxemia and dysregulated inflammation in malnourished mice. Strikingly, impaired intestinal barrier dysfunction primarily affected the caecum and colon.

The high caloric density of RUTF and RUSF is typically provided by lipids rich in omega-6 FAs.^(30,31)^ While providing needed calories, omega-6-rich lipids are pro-inflammatory and could fuel increased inflammation in malnourished children.^(30,31,32)^ In contrast, long-chain omega-3 polyunsaturated fatty acids (PUFAs), such as those found in fish oil, have anti-inflammatory effects.^(33)^ The recently published Codex Alimentarius^(34)^ and a review by Nienaber et al.^(35)^ provide valuable guidance on the inclusion of long-chain omega-3 PUFAs and the optimal ratio of omega-6 to omega-3 PUFAs in RUTF. In this study, we hypothesised that increasing the dietary intake of n-3 PUFAs would reduce malnutrition-related inflammation. We substituted fish oil for corn oil as the primary lipid source in the polynutrient-deficient diet to achieve a healthier ratio of dietary omega-6 to omega-3^(34,36,37)^ and evaluated the effects on systemic inflammation, intestinal mucosal barrier, and translocation of bacteria and bacterial LPS.

## Materials and methods

### Ethical considerations

The care experimental use of laboratory mice was in accordance with institutional and national regulations including the NIH Guide for the Care and Use of Laboratory Animals. The experimental protocol was reviewed and approved by the Institutional Animal Care and Use Committee (protocol 2107045) at the University of Texas Medical Branch (UTMB), Galveston, Texas, USA.

### Mouse diet

Weanling female BALB/C mice were obtained from Harlan Laboratories. Mice were grouped in cages of 5 and had free access to water. Mice were placed on one of three diets (formulated by Envigo, Envigo Teklad Diets, Madison WI) for 28 days: (i) a control mouse chow that contained normal protein, iron, and zinc and had corn oil as the lipid source (Envigo, TD.99103) (ii) an isocaloric but protein-, iron-, and zinc-deficient chow with corn oil as the lipid source (malnourished-corn oil; MN-CO) (Envigo TD.99075), or (iii) a chow identical to the MN-CO chow but formulated with fish oil in place of corn oil (MN-FO) (Envigo TD.160285). Details of control and MN-CO diet compositions have been published previously,^(26)^ and a comparison of the 3 diets is shown in Table 1. Mice were pair-fed every 48–72 hours and weighed every 7 days. MN-CO and MN-FO mice received 90% volume by weight of food that mice in the control group consumed. This was calculated by determining food consumption (grams/mouse/day) for the previous feeding period for control mice and then determining the necessary amount per cage for MN-CO and MN-FO mice. Mice were studied after 28 days on the diet.

**Table 1.** Composition of mouse chows Table 1 long description.

	Control	MN-CO	MN-FO
Protein (%)	17	3	3
Dextrose (g/kg)	643	807	807
Corn Oil (g/kg)	90	90	0
Fish Oil (g/kg)	0	0	90
Iron (ppm)	100	10	10
Zinc (ppm)	30	1	1
SFA (%FA)	14.8	14.8	20.5
MUFA (% FA)	26.2	26.2	25.1
PUFA (% FA)	59.0	59.0	54.5
Omega-3 (g/kg)	2.6	2.6	12.0
Omega-6 (g/kg)	44.9	44.9	29.0
Omega-6:Omega-3	17.5	17.5	2.4

### Collection of blood

Terminal blood collection was performed via cardiac puncture with a heparinised syringe with the mice under isofluorane anaesthesia, followed by immediate euthanasia by CO_2_ narcosis and cervical dislocation.

### Systemic LPS challenge

After 28 days on the control, MN-CO, or MN-FO diets, mice (4 or 5 mice per group) were challenged intraperitoneally with 4 mg/kg of LPS (*Escherichia coli* O55:B5, Sigma–Aldrich, L2880) in phosphate-buffered saline (PBS) or with PBS alone. Mice were given food and water *ad libitum*, and at 6 and 24 hours post-challenge, mice were weighed. After weighing at 24 hrs post-challenge, the mice were euthanised by CO_2_ narcosis and cervical dislocation.

### RNA isolation and RT-qPCR

Ileal samples were taken from the distal small intestine, cut into pieces, and stored in RNAlater Stabilisation Solution (Thermofisher) at −80°C. Colon samples were taken from the caecum and proximal colon. Thirty mg or less of intestine was homogenised in lysis buffer (Qiagen) + B-mercaptoethanol (Sigma) via bead mill (VWR 4-Place Mini Bead Mill Homogeniser) and spun down to pellet remaining tissue. RNA was extracted from the supernatant (RNeasy, Qiagen) and treated with DNAse (Turbo DNAse, Ambion). All RNA samples were quantified using a Thermo Scientific NanoDrop™ Spectrophotometer and stored at −80°C. cDNA was generated from each sample using the High Capacity cDNA Reverse Transcription kit (Applied Biosystems) (Veriti 96-Well Thermal Cycler, Applied Biosystems). Cytokine mRNA was quantified using 20–40 ng cDNA, SYBR Green Master Mix (ThermoFisher), and primers (Supplementary Table 1) on a Viia 7 Real-Time PCR System (Applied Biosystems). The fold change of gene expression was calculated with the delta CT method and 18S as the reference gene. The fold-change was calculated relative to the value in mice in the control group, or the uninfected control group in the *Citrobacter rodentium* infection studies.

### Quantification of bacterial burden in mouse tissue

Spleen, liver, and mesenteric lymph nodes (MLNs) were used to determine bacterial burden. The liver was cut into thirds, and the anterior portion of the middle section was collected. The spleen was cut in half, and the anterior half was collected. The MLN were collected and used for bacterial quantification. Spleen, liver, and MLN samples were stored in a 10x volume of PBS (ex. 50 mg tissue, 500 µl PBS) in a 2 ml Eppendorf tube. Samples were homogenised in the collection tube with a rubber pestle. Each sample was serially diluted out to 10^−6^ in PBS, generating a range of tissue concentrations from 10 mg/µl to 10 ng/µl. 50 µl aliquots of each dilution were plated on Brain Heart Infusion Agar (Sigma–Aldrich) plates and incubated aerobically at 37°C. Plates were incubated 3 days and colonies counted on both days 2 and 3. Colony counts and dilution values were used to calculate CFU/g for each sample.

### Determination of intestinal microbiota composition

To determine the microbiota composition in the MN-CO, MN-FO, and control mice, contents from the caecum were collected immediately after euthanasia and snap-frozen at −80°C. DNA was isolated from caecal matter using the QIAamp DNA Stool Mini Kit (QIAGEN) according to manufacturer’s protocol and shipped on dry ice to LCSciences (Houston, Texas, USA) for 16S rDNA V3+V4 sequencing and analysis. Bacterial DNA was amplified with primers targeted to the V3 and V4 regions of 16S rDNA. Sequencing adaptors and barcodes were added in further amplification steps, and the prepared library was sequenced on the MiSeq platform. Resultant paired-end reads were merged into tags and then grouped into clusters that represent a single operational taxonomic unit (OTU) (minimum 97% sequence similarity). Analysis of OTUs were conducted including measures of alpha diversity, beta diversity, and taxonomy annotation. Databases for taxonomy analysis included RDP (version date 2016.9.30) and NT-16S (version date 2016.10.29). Z scores for the proportions of genera were calculated using a Wilcoxon rank sum test.

### *Citrobacter rodentium* challenge

*Citrobacter rodentium* was grown overnight in Luria Broth at 37°C while shaking. Subcultures were made by diluting 200 µl of overnight culture in 10 mL LB and incubating at 37°C with shaking for 4 hours. Subcultures were pooled, washed with PBS, and resuspended in PBS. The culture density was measured via spectrophotometer and adjusted to 5 × 10^10^ bacteria/µL. Mice were orally gavaged with 200 µl, resulting in a dose of 1 × 10^10^ bacteria. Dilutions of the inoculum were plated on MacConkey agar (M7408, Sigma–Aldrich, Missouri, USA) to verify bacterial counts. Diets and monitoring were continued as before, with the exception that water bottles were replaced with HydroGel packs (ClearH_2_O, Maine, USA). Weight and at least two fresh faecal pellets per mouse were collected on days 0, 3, 5, 7, 10, and 14. Pellets were weighed and homogenised in PBS before dilution and plating on MacConkey agar. Colonies of *C. rodentium* were counted, and CFU/g of faecal matter was used to calculate bacterial burden. On day 14 post-gavage, mice were euthanised and the caecum tissue collected, washed, and stored in RNAlater at -80°C until RT-qPCR analysis.

### Statistical analysis

Comparisons between 2 groups were evaluated with two-tail Mann–Whitney U test for non-parametric data or two-tail unpaired t test for normally distributed data. Comparisons between more than 2 groups were evaluated with Kruskall-Wallis for non-parametric data or ANOVA for normally distributed dated with post hoc correction for multiple comparisons (Bonferroni or Tukey). Outliers were identified via the ROUT method with coefficient Q of 1%^(38)^ and removed from analysis where appropriate. All analyses were conducted using GraphPad Prism 10 for Mac OS X (Graphpad Software, San Diego California USA) or R 3.4.3. Data are presented in graphs as the mean and standard deviation of the group, or when the data were skewed, they are presented as the median with interquartile range, which is noted in the figure legends.

## Results

### Dietary fish oil promotes maintenance of body mass in malnourished mice

**Figure 1.**
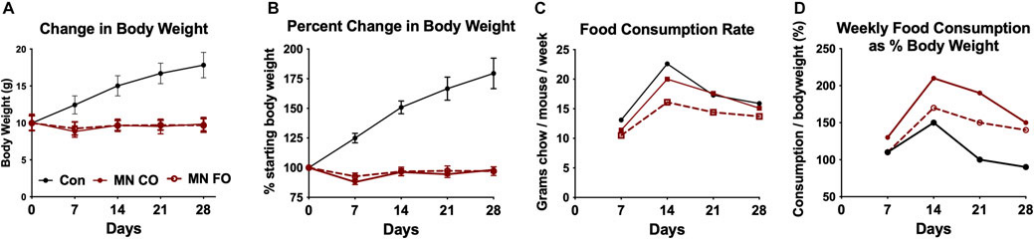
Figure 1 long description. Malnourished mice receiving dietary fish oil consumed less food while maintaining similar body weight to malnourished mice receiving corn oil. Weight change in grams of body weight **(A),** and as a percentage of starting weight **(B),** over the 28-day period on the experimental diets. Data are presented as the mean and standard deviation of 5 mice per group. **(C)** Food consumption rate (grams per mouse per week) over the 28-day period on the experimental diets. Data are presented as the mean of 5 mice per group. Food consumption was calculated for each cage (5 mice) by subtracting the weight of food remaining after 1 week from the weight of food at the start of the week and dividing by 5. **(D)** Weekly food consumption as a percent of body weight. Data are presented as the mean of 5 mice per group.

To determine the effect of dietary fish oil in malnourished mice, we monitored food consumption and body weight over the course of 28 days for mice fed a normal control diet with corn oil as the primary dietary lipid,^(25,26,27,28,29)^ or a diet deficient in protein, iron and zinc (malnourished; MN) that included either corn oil (MN-CO) or fish oil (MN-FO) as the primary dietary lipid. The nutrient analysis of the different diets is detailed in Table 1. Studies were performed in the first 28 days of the experimental diets because our previous studies indicated that mice fed a normal mouse chow (healthy controls) reached a weight plateau at approximately 28 days post-weaning.^(25,26,27,28,29)^ Replacement of corn oil with fish oil in the malnourished groups of mice had no effect on body weight over the course of the 28 days (Figure 1A and B). However, mice in the MN-FO group maintained similar weights and rate of weight change to mice in the MN-CO group while consuming significantly fewer grams of food per mouse per week (Figure 1C; *p* = 0.0461) and when food consumption was calculated as a percentage of body weight (Figure 1D; *p* = 0.0060). This suggests that FO-fed mice had either reduced energy expenditure, possibly from reduced inflammation as described below, or increased nutrient harvesting efficiency so that they maintained a similar weight to CO-fed mice while taking in fewer calories.

### Dietary fish oil reduces basal systemic inflammation in MN mice

**Figure 2.**
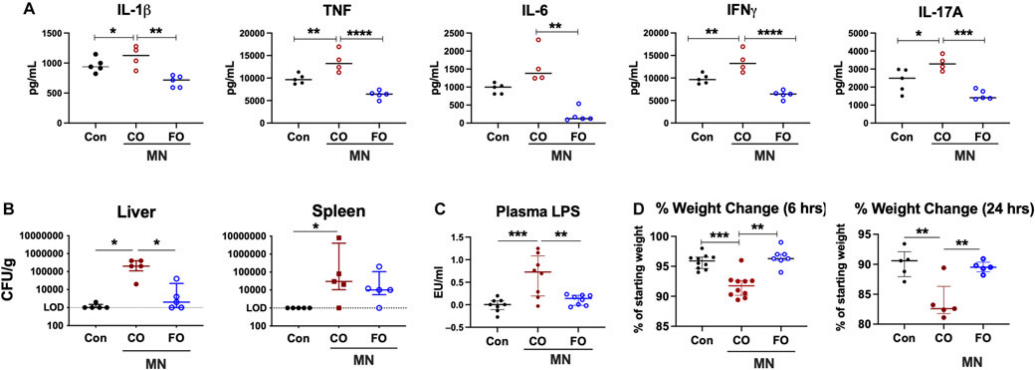
Figure 2 long description. Dietary fish oil reduced systemic inflammation, translocation of bacteria and bacterial LPS, and blunted the exaggerated physiological response to challenge with bacterial LPS in Malnourished Mice. **(A)** Plasma concentrations (pg/mL) of inflammatory cytokines (IL-1b, TNF, IL-6, IFNg, and IL-17A) measured by multiplex immunoassay after 28 days on the experimental diets. Shown as scatter plots of values from individual mice with a line at the median (*n* = 4–5 mice per group). **(B)** Burden of culturable aerobic bacteria in the liver and spleen measured after 28 days on the experimental diets. Data are shown as a scatter plot of CFU per gram tissue from individual mice (*n* = 5 per group) with the median and interquartile range. **(C)** Plasma LPS measured by bioassay in mice after 28 days on the experimental diets. Data are shown as a scatter plot of individual mice (*n* = 8 per group) with the median and interquartile range. **(D)** Mice after 28 days on the experimental diets were challenged with a sub-lethal dose of *E.coli* LPS by intraperitoneal injection. Scatter plots of individual mouse body weights (*n* = 7–10 per group) shown as percent of starting body weight with the median and interquartile range at 6 hrs and 24 hrs post-LPS challenge. (*p < 0.05 ** *p* < 0.01 ***p < 0.001 ****p < 0.0001).

We previously demonstrated increased basal inflammation and exaggerated inflammatory responses to inflammatory stimuli in malnourished children.^(22)^ This was recapitulated in mice on a diet deficient in protein, iron, and zinc with corn oil as the primary lipid source, compared to control mice on an isocaloric, nutrient-replete diet with corn oil as the primary lipid source.^(23)^ Therefore, mice on the isocaloric, nutrient-sufficient diet with corn oil are used as control mice for the studies presented here. Since dietary fish oil was found to reduce systemic inflammation in several disease models,^(33)^ we hypothesised that it would ameliorate baseline inflammation in our model of MAM. The level of inflammatory mediators in serum of MN-CO, MN-FO, and control mice was determined 28 days after initiation of the diet. As we described previously,^(23)^ the serum concentration of proinflammatory cytokines was increased in MN mice with corn oil as the lipid source (MN-CO) compared to mice on a normal diet with corn oil as the lipid source (Figure 2A). Malnourished mice that had the corn oil in the nutrient-deficient diet replaced with fish oil (MN-FO) had reduced serum inflammatory cytokines (IL-1β, TNF, IL-6, IFNγ, and IL-17A) relative to mice in the MN-CO group (Figure 2A).

### Dietary fish oil reduces translocation of culturable bacteria and bacterial LPS in MN mice

In our previous studies, malnutrition led to increased translocation of bacteria and bacterial LPS from the gut to the visceral organs.^(23)^ Here we similarly found that MN-CO mice had increased culturable bacteria in the liver and spleen compared to mice on the control diet. Mice in the MN-FO group had significantly less bacteria in the liver compared to mice in the MN-CO group (*p* = 0.016) but an insignificant decrease in bacteria in the spleen (*p* = 0.333) (Figure 2B). There were no differences in the bacterial load in the mesenteric lymph nodes among the three groups (data not shown). Mice in the MN-CO group also had increased plasma LPS compared to control mice, and the endotoxemia was significantly reduced in malnourished mice by inclusion of fish oil in the diet (Figure 2C). Collectively these data indicate that malnourished mice whose dietary lipid is rich in omega-6 PUFAs have impaired intestinal mucosal barrier function that allows increased translocation of bacteria and LPS, and that intestinal barrier function is improved with inclusion of lipids rich in omega-3 PUFAs.

### Dietary fish oil blunts the exaggerated physiological response to systemic challenge with bacterial LPS

We evaluated the effect of dietary fish oil on the physiological response to an inflammatory stimulus in MN mice. Because recurrent bacterial infections play a significant role in growth faltering of malnourished children,^(39,40,41,42,43)^ we mimicked this by challenging mice systemically with a sublethal dose of bacterial LPS. At six hours post-challenge, mice in the MN-CO group had a median weight reduction of 7.9% from their starting weight, which was significantly greater than the control group (*p* < 0.001) (Figure 2D). Mice in the MN-FO group lost a median of 3.8% from their starting weight, which was no different than the control mice (a median of 4.2% reduction from their starting weight) but significantly less than mice in the MN-CO group (*p* < 0.01). At 24 hours post-challenge, LPS-induced weight loss was also significantly improved in mice in the MN-FO group (Figure 2D). These data indicate that dietary fish oil (rich in omega-3 PUFAs) ameliorates the exaggerated physiological response observed in malnourished mice whose dietary lipid is rich in omega-6 PUFAs.

### Dietary fish oil improves intestinal immune defence and mucosal barrier function

**Figure 3.**
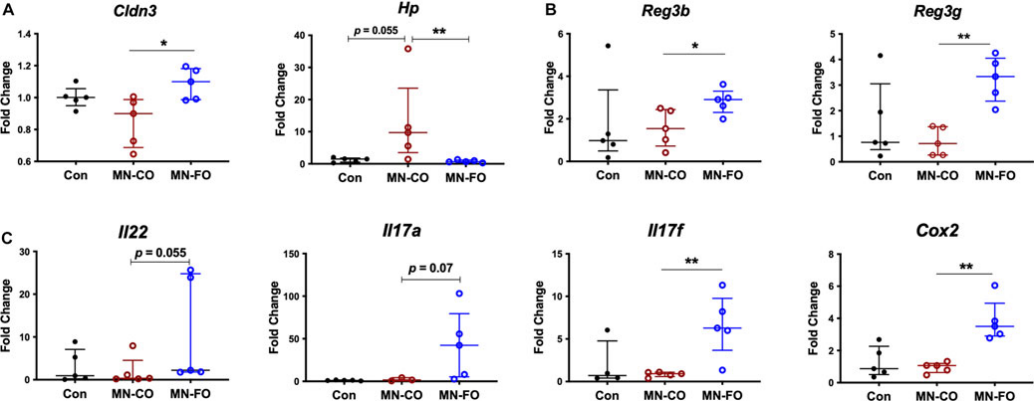
Figure 3 long description. Dietary fish oil improved intestinal barrier function and antimicrobial defence in Malnourished Mice**. (A)** mRNA expression of proteins involved in intestinal barrier function (Cldn3 and Hp). **(B)** mRNA expression of proteins involved in intestinal antimicrobial defence (Reg3b and Reg3g). **(C)** mRNA expression of inflammatory mediators involved in maintenance of intestinal mucosal integrity and antimicrobial defence (IL-22, IL-17A, IL-17F, and COX2). All data are shown as scatter plots of values from individual mice (*n* = 5 per group) with the median and interquartile range. (*p < 0.05 ** *p* < 0.01).

In this model of moderate acute malnutrition, we previously demonstrated that the increased translocation of bacterial LPS in malnourished mice was driven by intestinal dysbiosis with expansion of Gammaproteobacteria and contraction of Bacteroidetes, increased total faecal LPS biomass, and evidence of impaired intestinal barrier function.^(23)^ The finding that dietary fish oil reduced translocation of culturable bacteria and bacterial LPS suggested that it improved intestinal barrier function by directly or indirectly affecting the intestinal epithelium, antibacterial mucosal defence, or altering the intestinal microbiota. We evaluated each of these possibilities. Compared to mice in the MN-CO group, we found that mice in the MN-FO group exhibited increased mRNA of the tight junction protein *Cldn3* and a decrease in *Hp* mRNA (Figure 3A), which encodes the precursor of zonulin (a protein that promotes intestinal permeability by interacting with tight junction proteins).^(44)^ The increased *Cldn3* and the reduced *Hp* in the MN-FO mice indicate improved intestinal mucosal barrier. We also found that MN mice receiving dietary fish oil displayed increased mRNA levels of antimicrobial proteins *Reg3b* and *Reg3g* (Figure 3B). Transcripts of other inflammatory mediators that have a role in maintenance of intestinal barrier, including IL-22 (*p* = 0.055), IL-17A (*p* = 0.07), IL-17F (*p* < 0.01), and COX2 (*p* < 0.01) were increase in mice in the MN-FO group compared to the MN-CO group (Figure 3C). Collectively, these data indicate that dietary fish oil improves the impaired intestinal immune defence and mucosal barrier function in malnourished mice.

### Dietary fish oil improves caecal microbiota diversity and promotes a healthier microbiota composition

**Figure 4.**
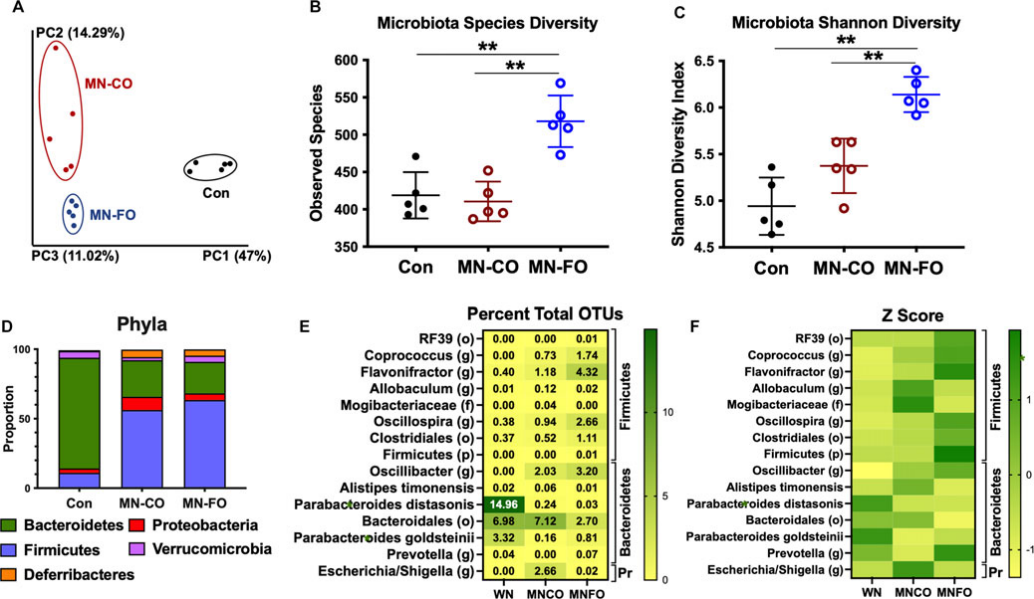
Figure 4 long description. Dietary fish oil increased caecal microbiota diversity and altered the microbiota composition to promote intestinal health in malnourished mice**. (A)** Principal component analysis of the microbiota composition demonstrating distinct separation of the control (Con), MN-CO, and MN-FO groups. Individual symbols represent the microbiota composition of each mouse (n=5 mice per group). **(B, C)**. Microbiota diversity was calculated based on observed OTUs **(B)** or Shannon Diversity index **(C)** for all groups (n=5 mice per group). **(D)** Mean phyla proportions in caecal microbiota from mice in the control (Con), MN-CO, and MN-FO groups. **(C, D)** Heatmaps of OTUs present at significantly different (*p* < 0.05) proportions in mice in the MN-CO and MN-FO groups displayed as % of population **(C)** or as *z* score **(D)**. *z* scores were calculated using mean and SD for each OTU. Heatmaps are arranged by phyla (Firmacutes, Bacteroidetes, and Proteobacteria [Pr]) and in descending order of statistical significance.

The microbiota compositions of the caecal contents of mice in the control, MN-CO, and MN-FO groups, determined by 16S rRNA sequencing, were clearly distinguished by principal component analysis (Figure 4A). Microbiota of mice in the MN-FO group had significantly greater diversity than mice in either control or MN-CO groups, measured by number of observed OTUs (Figure 4B) or Shannon index (Figure 4C). As we previously reported in this model, MN mice had significant contraction of the phyla Bacteroidetes and expansion of Firmacutes and Proteobacteria. We observed a two-fold decrease in proportion of Proteobacteria in mice in the MN-FO group (4.9%) compared to the MN-CO group (9.5%) (Figure 4D; *p* = 0.008), driven almost entirely by reduction of an OTU assigned to *Escherichia*/*Shigella* (Figures 4E and F). There were 15 additional OTUs whose percentage differed significantly between mice in the MN-CO and MN-FO groups (Figures 4D and E). Dietary fish oil significantly increased intestinal bacteria of the genera *Flavonifractor*, *Coprococcus*, *Oscillospira*, and *Oscillibacter*, each of which is generally associated with beneficial effects on metabolism and gut health (Figures 4E and F).

### Dietary fish oil does not compromise intestinal defence against *Citrobacter rodentium* infection

**Figure 5.**
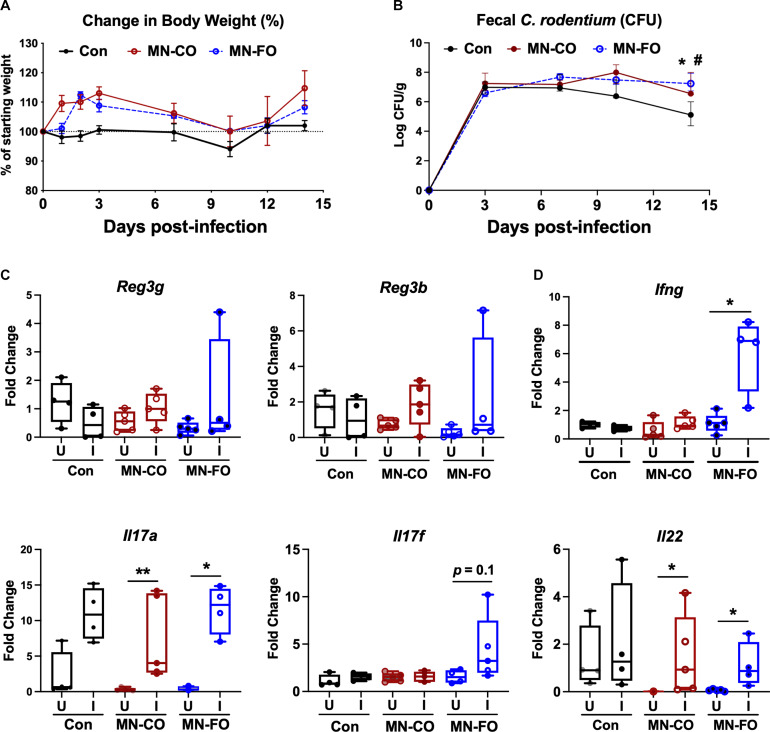
Figure 5 long description. Dietary fish oil does not compromise defence against intestinal infection with *Citrobacter rodentium*. Mice in the Control (Con), MN-CO, and MN-FO groups (*n* = 5 mice per group) after 28 days on the experimental diets were infected with *C. rodentium* by oral gavage and observed for 14 days. **(A)** Weight change (mean and SD) are plotted over the course of the 14 days. **(B)** Median and IQR of log colony-forming units (CFU)/g of faecal matter collected from Control (Con), MN-CO, and MN-FO groups, at 0, 3, 7, 10, and 14 days post-infection. *p < 0.05 for comparison of MN-CO to Con; # *p* < 0.05 for comparison of MN-FO to Con. **(C, D)** mRNA expression of Reg3b and Reg3g proteins involved in intestinal antimicrobial defence **(C)** and cytokines involved in intestinal antimicrobial defence and maintenance of mucosal barrier **(D)**, in proximal colon tissue collected 14 days after *C. rodentium* infection. Data are shown as boxed scatter plots of values from individual mice (*n* = 4 per group) with the median and interquartile range. (*p < 0.05, ** *p* < 0.01).

*Citrobacter rodentium* is a natural intestinal pathogen of mice that causes infection similar to that of the closely related human pathogens enteropathogenic and enterohemorrhagic *Escherichia coli.* ^(45)^ There is concern that blunting of the inflammatory response, impaired LPS detoxification, and modulation of T cell and macrophage function by dietary FO^(46,47)^ could lead to impaired host defence against infectious pathogens. Therefore, we determined the impact of dietary fish oil on control of infection with an enteric pathogen. We infected mice in the control, MN-CO, and MN-FO groups with *Citrobacter rodentium* after 28 days on the experimental diets. Both MN-CO and MN-FO mice gained weight over the initial 3 days post-infection while control mice remained static (Figure 5A). All mice experienced a temporary weight loss between 3 and 14 days that coincided with the timing of the immune response as typically observed in mouse models.^(45)^ The *C. rodentium* burden in faecal pellets was similar between control, MN-CO, and MN-FO groups until day 14, at which point the burden in control mice dropped significantly below that of MN-CO and MN-FO groups. There were no significant differences in bacterial burden or weight change between MN-CO and MN-FO mice (Figure 5B). There were no significant differences in the mRNA expression of Reg3g and Reg3b in the proximal colon (Figure 5C). All groups responded to *C. rodentium* infection with increased intestinal *Il17a* and *Il22* expression; however, only mice in the MN-FO group increased expression of *Ifng* in response to oral *C. rodentium* challenge (Figure 5D).

## Discussion

The findings presented here show that replacement of corn oil (high in omega-6 PUFAs) with fish oil (high in omega-3 PUFAs) in a nutrient-deficient diet can abrogate malnutrition-related intestinal dysbiosis, translocation of bacteria and bacterial LPS across the intestinal mucosa, and systemic inflammation. Importantly, MN mice that received dietary FO mice were better able to tolerate systemic challenge with LPS. Therefore, incorporation of omega-3 LC-PUFAs into MAM treatment programmes could be beneficial in correcting energy deficits while also mitigating the increased systemic inflammation found in children with MAM.

In this study, we reasoned that increased dietary omega-3 LC-PUFAs in mice with MAM would have a beneficial effect in blunting the malnutrition-associated inflammation. Incorporation of long-chain n-3 PUFAs into cellular membranes inhibits TLR4 signalling^(48)^ and reduces production of inflammatory eicosanoids.^(33)^ Resolvins and protectins, lipid mediators produced from DHA and EPA, encourage resolution of inflammation^(49)^ We found that overall, fish oil consumption reduced translocation of intestinal bacteria and bacterial LPS. This may be the result of improved intestinal dysbiosis (reduced Proteobacteria) or improved intestinal mucosal barrier function, or both.

Curiously, dietary FO enabled MN mice to maintain the same weight as MN mice while consuming fewer calories. This suggests that they are better able to harvest nutrients from their food and/or expend less energy. The inflammatory cytokine data suggest that dietary FO enables malnourished mice to conserve energy by reducing systemic inflammation. A previous study of mice on a starvation diet found that supplementation with 4% FO improved survival and appearance by normalising neurochemical signalling related to satiety.^(50)^ Thus, it is possible that in our model malnourished mice given FO consume less food without increased wasting due to the satiety-inducing effects of FO on neurochemical signalling or conservation of energy from reduced systemic inflammation. Future studies in metabolic chambers could address this question.

Compared to MN mice, MN-FO mice had reduced translocation of gut bacteria to the liver but similar levels of culturable bacteria in the MLN and spleen. As we described previously, serum LPS bioactivity was dramatically increased in mice on the MN-CO diet compared to controls, but dietary fish oil abrogated the malnutrition-related LPS translocation. This outcome may be driven by (i) restored intestinal mucosal barrier function, (ii) improved MLN barrier function so that fewer bacteria escape the LN, and/or (iii) reduced LPS mass in the intestine related to microbiota changes (reduced Proteobacteria that bear high-potency LPS). The increase in transcripts for intestinal barrier-enhancing proteins, antimicrobial peptides, and inflammatory modulators known to promote intestinal barrier support the idea that fish oil promotes mucosal defence. The Reg3 proteins are an important barrier against microbial invasion by intestinal commensals or pathogens. Reg3γ is a secreted antimicrobial peptide that primarily target the peptidoglycan cell wall of Gram-positive bacteria,^(51,52)^ while Reg3β appears to bind directly to LPS and has antimicrobial activity against Gram-negative bacteria.^(53,54)^ They also regulate the balance of the gut microbiota^(55)^ and promote regeneration and healing of tissue damage, making them important for maintaining intestinal barrier function. The Th17 cytokines have a significant role in maintaining the intestinal mucosal barrier.^(56)^ Both IL-22^(57)^ and the IL-17 family^(58)^ promote the expression of the Reg3 antimicrobial peptides. COX2 also has a role in maintaining intestinal mucosal integrity.^(59)^ Collectively, these data suggest that replacing dietary omega-6-rich corn oil with omega-3-rich fish oil in mice on a diet deficient in protein, iron, and zinc improved intestinal barrier function and reduced translocation of bacteria and bacterial LPS.

The changes in the intestinal microbiota of MN mice that received fish oil may drive downstream reduced inflammation. While fish oil consumption significantly altered the microbiota in MN mice, it did not change the microbiota of control mice. Thus, while the changes observed in the microbiota of MN mice that received FO may be beneficial, transition to a normal microbiota and recovery from MAM will also require correction of the nutrient deficits (in this case low protein, iron and zinc). One of the most striking findings is that the MN-FO caecal microbiota exhibited significantly greater alpha diversity than microbiotas of WN or MN mice. This increased diversity of OTUs likely indicates an increased diversity of bacterial genes and functional groups which may afford increased resiliency to perturbation and ability to harvest nutrients from different foods.^(60)^ Despite the differences in diversity, only 15 OTUs differed significantly in proportion in the MN-FO and MN groups. At the phyla level, MN-FO mice had a significant reduction of proportion of Proteobacteria compared to MN mice. This was largely driven by a significant decrease in an OTU attributed to the *Escherichia/Shigella* group. Previous studies in this model of MAM identified expansion of Gammaproteobacteria as a driver exaggerated systemic inflammation.^(23)^ Studies in humans and other models have also demonstrated the proinflammatory nature of the Gammaproteobacteria [reviewed in].^(61,62)^ The aforementioned study by Ghosh et al.^(47)^ also observed a decrease in Enterobacteriaceae proportion in FO-supplemented mice. Another study by Kaliannan et al.^(63)^ of mice with elevated tissue omega-3 PUFA levels also observed a reduction in proportion of Proteobacteria. The reduction of Proteobacteria and in particular Escherichia/Shigella may reduce systemic inflammation and inflammatory response in MN-FO mice by reducing the amount of intestinal and circulating endotoxin.^(23)^ Besides reducing the proinflammatory intestinal Proteobacteria, dietary fish oil increased intestinal bacteria of the genera *Flavonifractor*, *Coprococcus*, *Oscillospira*, and *Oscillibacter*, each of which is generally associated with beneficial effects on metabolism and gut health. Bacteria in the genus *Flavonifractor*, particularly *F. plautii,* metabolise plant-derived flavonoids and reduce intestinal inflammation.^(64)^ *Coprococcus* spp.^(65)^ and *Oscillospira* spp.^(66)^ are significant producers of butyrate and other short-chain fatty acids (SCFAs) that serve as the primary energy source for colon cells. SCFAs also help maintain the integrity of the gut mucosa and have anti-inflammatory effects. *Oscillibacter* can metabolise cholesterol and has immunomodulatory effects that can mitigate inflammation.^(67)^

Fish oil consumption can reduce inflammatory cytokine production in neutrophils, monocytes, and macrophages,^(68)^ so has the potential to compromise host defence.^(46,47)^ In this study dietary fish oil did not compromise defence against infection with *C. rodentium*, an intestinal pathogen that can cause severe colitis and growth faltering in mice.^(69)^ In fact, intestinal Ifng, Il17, and Il22 responses, which are critical to defence against *C. rodentium* infection,^(55,56,70)^ were as strong or stronger in the MN-FO group compared to mice in the control and MN-CO groups. This is most likely due to the beneficial effect of n-3 fatty acids on intestinal epithelial integrity.^(71)^ While both malnourished groups exhibited delayed clearance compared to control mice no clinical consequences were observed. Ghosh et al. also found that addition of FO to a high-fat diet in mice reduced intestinal damage and translocation during *C. rodentium* infection.^(47)^ We observed no negative consequences of fish oil consumption in our model, adding to the literature on the safety of omega-3 LC-PUFAs as a nutritional intervention.

Our findings have several implications for treatment of acute malnutrition. Children with MAM often experience treatment failure or relapse.^(72)^ Most trials of new RUTF and RUSF formulations have focused on weight gain, safety, and cost.^(32,72,73)^ A recent review of the effect of varied fatty acid profiles in RUTF^(35)^ highlighted that only the study by Jones et al.^(30)^ assessed effects of fish oil supplementation on inflammation. They found no major differences in soluble inflammatory markers in plasma. Omega-3 long-chain PUFAs are critical for early child development^(74)^ but are reduced in children with MAM^(75)^ and in traditional RUTFs.^(32)^ Since inflammation contributes to the pathophysiology of malnutrition,^(22,23,76,77, 78)^ low levels of omega-3 PUFA were inversely correlated with markers of inflammation in children with MAM,^(75)^ and plasma omega-3 FAs decreased during treatment with standard RUTF formulations, concern about the high omega-6 to omega-3 ratio in RUTF has been raised.^(32,35)^ Two clinical trials have been conducted that incorporated omega-3 PUFAs in treatment of malnourished children.^(30,32,79)^ Both used RUTFs with increased levels of omega-3 alpha-linoleic acid (ALA), a precursor of DHA and EPA. However, increased dietary ALA did not improve DHA status in either study, likely due to the poor conversion rate of ALA to DHA,^(32)^ but DHA supplementation alongside the ALA-high RUTF was effective in raising blood DHA levels.^(30)^ Fish oil or purified n-3 PUFAs are candidates for inclusion in next-generation RUTFs and RUSFs to provide longer-lasting nutritional recovery by simultaneously correcting nutrient deficiencies and the pathophysiological systemic inflammation and intestinal dysbiosis. Furthermore, DHA is a major structural component of the brain cortex, and RUTF with added DHA showed to improve cognition compared with standard RUTF.^(80)^ A meta-analyses suggest that supplementation of DHA and DEA to achieve an Omega-3 Index >6% is associated with improved cognitive function in children and adolescents.^(81)^ Omega-3 has had demonstrated efficacy demonstrated in protection from neuroinflammation,^(81)^ and treatment of inflammatory skin conditions^(82)^ and inflammatory bowel disease.^(33,83)^

There are several limitations to this study. We completely substituted fish oil for corn oil in the diet to achieve the primary goal of a ratio of dietary omega-6 to omega-3 PUFAs similar to what is recommended for human health.^(34,36,37)^ While the positive effects we found with dietary fish oil are provocative, this is not a realistic approach, and the findings cannot be extrapolated directly to treatment of children with MAM. To mimic incorporation of fish oil into a therapeutic supplement, mice would need to be malnourished before initiating the dietary fish oil. Nevertheless, study of this model enabled initial identification of the effects of fish oil consumption in a model of MAM. While we investigated the effects of dietary fish oil on intestinal barrier function, microbiota composition, and host defence, additional studies to define broader physiological and metabolic effects would be revealing. We did not determine levels of DHA and EPA and their anti-inflammatory metabolites, but doing so would help elucidate the mechanisms by which fish oil impacts metabolism and immunity. Studies of body composition could determine if energy is stored differently. Finally, tests of different quantities of fish oil given at various times in the course of this model of MAM should be conducted to determine the optimal amount and timing for improving inflammatory and growth metrics.

## Conclusions

Increased dietary intake of fish oil in a mouse model of acute malnutrition reduced systemic inflammation, improved intestinal dysbiosis and intestinal barrier function, and reduced translocation of bacteria and bacterial LPS. Dampening inflammation through dietary omega-3 did not compromise host defence against an intestinal pathogen. Collectively, these data suggest dietary long-chain omega-3 PUFAs could be beneficial in the prevention or treatment of malnutrition. Future studies should assess the efficacy of supplementing dietary long-chain omega-3 PUFAs in children with malnutrition.

## Supporting information

10.1017/jns.2026.10114.sm001 Patterson et al. supplementary material Patterson et al. supplementary material

## Data Availability

Data will be made available on request.

## Supplementary material

The supplementary material for this article can be found at https://doi.org/10.1017/jns.2026.10114.

## Table 1. Long description

The table compares the composition of three different mouse chow diets across various nutritional components. It has four rows and eleven columns. The columns are labeled Control, MN-CO, and MN-FO, representing different diet types. The rows list the nutritional components: Protein percentage, Dextrose in grams per kilogram, Corn Oil in grams per kilogram, Fish Oil in grams per kilogram, Iron in parts per million, Zinc in parts per million, Saturated Fatty Acids percentage of fatty acids, Monounsaturated Fatty Acids percentage of fatty acids, Polyunsaturated Fatty Acids percentage of fatty acids, Omega-3 in grams per kilogram, Omega-6 in grams per kilogram, and the Omega-6 to Omega-3 ratio. The Control diet contains 17 percent protein, 643 grams per kilogram dextrose, 90 grams per kilogram corn oil, no fish oil, 100 parts per million iron, 30 parts per million zinc, 14.8 percent saturated fatty acids, 26.2 percent monounsaturated fatty acids, 59 percent polyunsaturated fatty acids, 2.6 grams per kilogram Omega-3, 44.9 grams per kilogram Omega-6, and an Omega-6 to Omega-3 ratio of 17.5. The MN-CO diet contains 3 percent protein, 807 grams per kilogram dextrose, 90 grams per kilogram corn oil, no fish oil, 10 parts per million iron, 1 part per million zinc, 14.8 percent saturated fatty acids, 26.2 percent monounsaturated fatty acids, 59 percent polyunsaturated fatty acids, 2.6 grams per kilogram Omega-3, 44.9 grams per kilogram Omega-6, and an Omega-6 to Omega-3 ratio of 17.5. The MN-FO diet contains 3 percent protein, 807 grams per kilogram dextrose, no corn oil, 90 grams per kilogram fish oil, 10 parts per million iron, 1 part per million zinc, 20.5 percent saturated fatty acids, 25.1 percent monounsaturated fatty acids, 54.5 percent polyunsaturated fatty acids, 12 grams per kilogram Omega-3, 29 grams per kilogram Omega-6, and an Omega-6 to Omega-3 ratio of 2.4.

Navigate back to Table 1..

## Figure 1. Long description

The image contains four line graphs labeled A, B, C, and D. Graph A shows the change in body weight in grams over 28 days for three groups: control, MN CO, and MN FO. Graph B displays the percent change in body weight from the starting weight over the same period. Graph C illustrates the food consumption rate in grams per mouse per week. Graph D presents weekly food consumption as a percentage of body weight. Each graph includes data points with error bars representing the mean and standard deviation of five mice per group. The control group shows a steady increase in body weight, while the MN CO and MN FO groups maintain similar body weights despite differences in food consumption rates and percentages.

Navigate back to Figure 1..

## Figure 2. Long description

The image contains four sets of scatter plots. The first set (A) shows plasma concentrations of inflammatory cytokines (IL-1b, TNF, IL-6, IFNg, and IL-17A) in malnourished mice after 28 days on experimental diets. Each plot includes values from individual mice with a line at the median. The second set (B) displays the burden of culturable aerobic bacteria in the liver and spleen, measured as CFU per gram of tissue, with scatter plots showing individual mice data, median, and interquartile range. The third set (C) presents plasma LPS levels measured by bioassay, with scatter plots of individual mice data, median, and interquartile range. The fourth set (D) illustrates the percentage of starting body weight of mice after a sub-lethal dose of E.coli LPS challenge, with scatter plots showing individual mouse body weights, median, and interquartile range at 6 and 24 hours post-challenge. Statistical significance is indicated with asterisks.

Navigate back to Figure 2..

## Figure 3. Long description

The image contains three sets of scatter plots labeled A, B, and C, each showing mRNA expression levels of different proteins and inflammatory mediators in malnourished mice. Each set compares three groups: control (Con), MN-CO, and MN-FO, with five mice per group. Plot A shows the expression of Cldn3 and Hp, which are involved in intestinal barrier function. Plot B displays the expression of Reg3b and Reg3g, which are involved in intestinal antimicrobial defense. Plot C illustrates the expression of inflammatory mediators IL-22, IL-17A, IL-17F, and COX2, which are crucial for maintaining intestinal mucosal integrity and antimicrobial defense. Each scatter plot includes individual data points, the median, and the interquartile range. Significant differences are indicated with asterisks, where *p < 0.05 and **p < 0.01. The data suggest that dietary fish oil (MN-FO) affects the expression of these proteins and mediators, potentially improving intestinal barrier function and antimicrobial defense in malnourished mice.

Navigate back to Figure 3..

## Figure 4. Long description

The image contains multiple graphs analyzing microbiota diversity and composition in malnourished mice fed different diets. The first graph (A) is a principal component analysis showing distinct separation of microbiota composition among control (Con), MN-CO, and MN-FO groups, with individual symbols representing each mouse. The second and third graphs (B and C) are scatter plots displaying microbiota diversity calculated by observed operational taxonomic units (OTUs) and Shannon Diversity index, respectively, for all groups. The fourth graph (D) is a bar chart showing mean phyla proportions in caecal microbiota from mice in the control, MN-CO, and MN-FO groups. The fifth and sixth graphs (E and F) are heatmaps of OTUs present at significantly different proportions in mice in the MN-CO and MN-FO groups, displayed as percentage of population and z score, respectively. The heatmaps are arranged by phyla and in descending order of statistical significance. All values are approximated.

Navigate back to Figure 4..

## Figure 5. Long description

The image contains four graphs analyzing the effects of different diets on mice infected with Citrobacter rodentium. The first graph is a line graph showing the percentage change in body weight over 14 days post-infection for three groups: Control (Con), MN-CO, and MN-FO. The second graph is a line graph displaying the log colony-forming units (CFU) per gram of fecal matter collected at 0, 3, 7, 10, and 14 days post-infection for the same three groups. The third set of graphs consists of box plots showing the fold change in mRNA expression of Reg3g and Reg3b proteins involved in intestinal antimicrobial defense in proximal colon tissue collected 14 days after infection. The fourth set of graphs consists of box plots showing the fold change in mRNA expression of cytokines involved in intestinal antimicrobial defense and maintenance of the mucosal barrier. Each graph includes data from individual mice with the median and interquartile range indicated. Significant differences are marked with asterisks and hashtags. All values are approximated.

Navigate back to Figure 5..
