# Supplementary material for: Dietary fish oil improves intestinal barrier function and intestinal microbiota composition and reduces systemic inflammation in a mouse model of moderate acute malnutrition
Source: J Nutr Sci. 2026 Jun 15;15:e43. doi: 10.1017/jns.2026.10114 (PMC13279968; doi:10.1017/jns.2026.10114)
Supplement: Patterson et al. supplementary material [file S2048679026101141sup001.pdf]

Supplementary Table 1 qRT-PCR primers used in this study

Gene Target	Primer	Sequence (5'-3')
IL22	IL-22 For	AAACTGTTCCGAGGAGTCAGTGCT
	IL-22 Rev	GCTGAGCTGATTGCTGAGTTTGGT
IL17a	IL17A For	ACCAGCTGATCAGGACGCGC
	IL17A Rev	CCAGGCTCAGCAGCAGCAACA
Il17f	IL17F For	TGCTACTGTTGATGTTGGGAC
	IL17F Rev	AATGCCCTGGTTTTGGTTGAA
Hp	Hp For	GCTATGTGGAGCACTTGGTTC
	Hp Rev	CACCCATTGCTTCTCGTCGTT
Reg3b	Reg3b For	ACTCCCTGAAGAATATACCCTCC
	Reg3b Rev	CGCTATTGAGCACAGATACGAG
Reg3g	Reg3g For	ATGCTTCCCCGTATAACCATCA
	Reg3g Rev	GGCCATATCTGCATCATACCAG
Cldn3	Cldn3 For	ACCAACTGCGTACAAGACGAG
	Cldn3 Rev	CAGAGCCGCCAACAGGAAA
18S	18S For	TTAGAGTGTTCAAAGCAGGCCCGA
	18S Rev	TCTTGGCAAATGCTTTCGCTCTGG

For, Forward primer; Rev, Reverse primer
